# Analysis of Sequential Pneumococcal Vaccination Coverage in the Elderly Resident Population of the Viterbo Local Health Authority from 2018 to 2023

**DOI:** 10.3390/vaccines13080807

**Published:** 2025-07-30

**Authors:** Andrea Bongiovanni, Giulia Santolini, Francesco Vairo, Francesco Corea, Silvia Aquilani, Chiara de Waure

**Affiliations:** 1 Department of Prevention, Local Health Authority of Viterbo, Via Enrico Fermi 15, 01100 Viterbo, Italy; andrea.bongiovanni@asl.vt.it (A.B.); francesco.corea@asl.vt.it (F.C.); silvia.aquilani@alice.it (S.A.); 2 National Institute for Infectious Diseases “Lazzaro Spallanzani”, Istituto di Ricovero e Cura a Carattere Scientifico—IRCCS, Via Portuense 292, 00149 Rome, Italy; francesco.vairo@inmi.it; 3 Department of Public Health and Infectious Diseases, Sapienza University of Rome, Piazzale Aldo Moro 5, 00185 Rome, Italy; giulia.santolini@uniroma1.it; 4 Department of Medicine and Surgery, University of Perugia, Piazzale Settimio Gambuli 1, 06156 Perugia, Italy

**Keywords:** pneumococcal vaccination, elderly immunization, vaccination coverage, immunization trends

## Abstract

Background: Pneumococcal disease is a significant health burden, particularly among older adults and individuals with chronic conditions. Sequential pneumococcal vaccination (PCV13 followed by PPSV23) has been recommended in Italy since 2017 for its demonstrated efficacy, safety, and cost-effectiveness in preventing invasive pneumococcal disease (IPD). Nevertheless, limited data are available on the sequential pneumococcal vaccination coverage in Italy. This study aimed to evaluate the coverage and trends of sequential pneumococcal vaccination among individuals who turned 65 years old within the Viterbo Local Health Authority between 2018 and 2023. Methods: A retrospective cohort study was conducted using data from the Regional Vaccination Registry (AVR), a comprehensive digital vaccination dataset. Vaccination coverage was calculated based on individuals completing the sequential pneumococcal vaccination within two years after turning 65 years old. Trends as well as subgroup variations based on sex, citizenship, district of residence, and municipality size were analyzed. Results: Among 27,657 individuals who turned 65 years of age during the study period, only 2.32% completed the sequential pneumococcal vaccination. Coverage increased steadily from 2018 (0.60%) to a peak in 2020 (3.27%), followed by a plateau and a decline in 2023 (2.53%). Coverage varied across demographic and geographic subgroups: females (2.58%) had higher coverage than males (2.04%), Italian citizens (2.45%) exceeded foreign residents (0.64%), and residents in District C (3.03%) led over District A (1.08%). Smaller municipalities (≤10,000 inhabitants) showed higher coverage (2.52%) than larger ones (1.98%). Conclusions: Adherence to sequential pneumococcal vaccination has been very low throughout the considered study period. This is highly relevant information to consider in the view of new available pneumococcal vaccines for immunization of the elderly. Furthermore, geographic and demographic differences highlight the need for targeted public health interventions.

## 1. Introduction

Pneumococcal disease is a significant cause of morbidity and mortality, especially among the elderly and individuals with specific underlying diseases [1]. In 2022, more than 17,700 cases of invasive pneumococcal disease (IPD) were reported in the European Union/European Economic Area, with an overall incidence of 5.1 cases per 100,000 inhabitants and a rate of 12.6 per 100,000 among adults aged 65 and over [2]. In Italy, the national surveillance system recorded an increasing trend in recent years, with an incidence of IPD reaching 3.02 cases per 100,000 inhabitants in 2023 overall, and 7.45 per 100,000 in the population aged ≥65 years [3].

The introduction of multivalent pneumococcal conjugate vaccines (PCVs) has decreased mortality in children under five, with estimated global pneumococcal deaths declining from 600,000 in 2000 to 294,000 in 2015 [4], and an impact on carriage of pneumococcal serotypes targeted by PCVs [5] in the overall population.

PCV’s effectiveness in preventing pneumococcal disease in children has been documented, initially for 7-valent PCV [6] and then for higher-valency vaccines such as 10-valent PCV [7] and 13-valent PCV [8,9]. Vaccination programs for children have also led to indirect protection of older adults via herd immunity [10], but based on the considerable and persisting burden of disease in the elderly, there has been a push towards targeted vaccination in this age group.

In Italy, despite the implementation of pneumococcal vaccination in Italian newborns starting from the beginning of the twentieth century, IPD remains the most common type of invasive bacterial disease [3], with regional disparities highlighting the need for enhanced public health interventions [11]. The Italian National Immunization Programme (NIP) 2012–2014 [12] recommended pneumococcal vaccination to individuals at higher risk independently by age, and the following NIP 2017–2019 [13] extended the recommendations to the elderly population, advising them to be vaccinated with PCV13 followed by 23-valent pneumococcal polysaccharide vaccine (PPSV23). The latter has been available for many years before the approval of PCV7 and is widely used to provide protection in adults and high-risk populations. PPSV23 targets a broader spectrum of pneumococcal strains but does not induce as strong an immune memory response as the conjugate vaccines do and is not able to elicit a protective immune response in children under 2 years of age. PPSV23 has been an important tool for the prevention of pneumococcal diseases in individuals at higher risk and was proven effective in preventing IPD and community-acquired pneumonia in particular [14].

More recently, in 2022, the 15-valent pneumococcal conjugate vaccine (PCV15) was approved in Italy for adults first [15] and for pediatric use later [16]. PCV15 includes two additional serotypes (22F and 33F) compared to PCV13 and shows a stronger response to serotype 3, which remains one of the leading causes of IPD in older adults [3]. Another conjugate vaccine, the 20-valent pneumococcal conjugate vaccine (PCV20), was approved for adults in 2021 [17] and for children in 2023 [18]. It includes additional serotypes not covered by PCV13 and PCV15, offering enhanced and more comprehensive coverage.

Based on the new available PCVs, the CDC [19] recommends administering PCV15, PCV20, or PCV21—the last PCV to become available, which covers eight serotypes not included in any of the other currently available vaccines [20]—to all adults 50 years or older who have never received any PCV or with an unknown vaccination history. The recommendations also foresee administering a follow-up dose of PPSV23 if PCV15 is used.

Italian NIP 2023–2025 [21], the latest and most current version, does not provide indications about the use of different available vaccines for vaccination of the elderly. Nevertheless, for practical reasons and to simplify vaccination management, many regions in Italy have started implementing protocols favoring the use of PCV20.

For an appropriate deployment of elderly pneumococcal vaccination, data on coverage are needed to both better understand which strategies can be implemented and monitor the vaccination campaign. Nevertheless, comprehensive and updated information on pneumococcal vaccination coverage is available for the pediatric population only, with national data showing a coverage rate of 91.73% and 94.38% in 2022 and 2023, respectively [22]. In contrast, knowledge about adults at risk and elderly vaccination coverage remains limited. In the Emilia-Romagna region, coverage with at least one dose of a PCV among cohorts born between 1952 and 1958 is reported to be 36.1% [23]. However, to the best of our knowledge, no data are available regarding adherence to the sequential pneumococcal vaccination.

Starting in 2018, the Lazio region implemented sequential pneumococcal vaccination to offer the elderly broader protection against pneumococcal diseases. With this research, we aim to monitor the vaccination strategy launched in 2018 by evaluating the coverage of sequential pneumococcal vaccination among individuals turning 65 years old in the Local Health Authority (LHA) of Viterbo, a province of the Lazio region of Italy.

## 2. Materials and Methods

### 2.1. Study Setting

**Figure 1:**
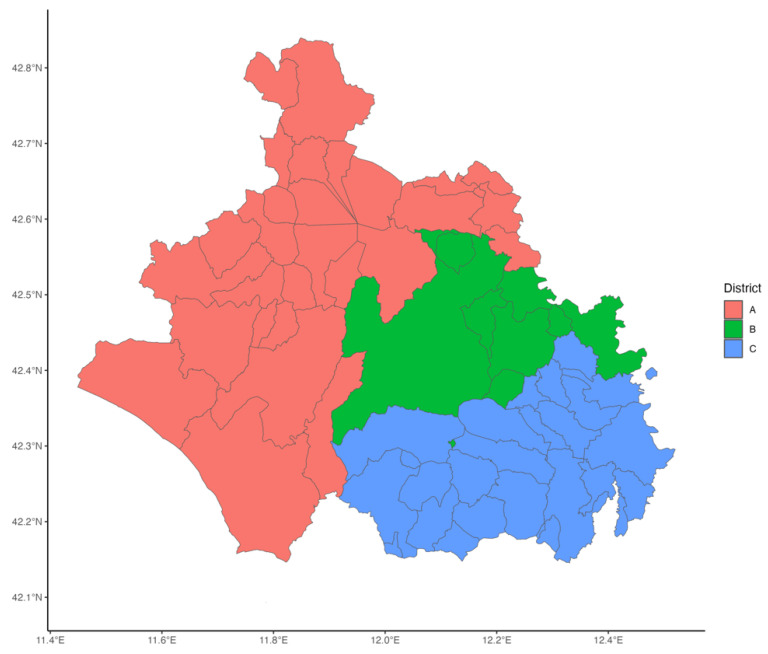
Geographic distribution of districts within Viterbo LHA.

The study was conducted within Viterbo LHA, which encompasses the city of Viterbo and 59 municipalities within Viterbo province. The LHA is administratively divided into three districts: A, B, and C, representing distinct territorial subdivisions for healthcare service delivery. These districts are illustrated in Figure 1. The LHA features vaccination centers specifically dedicated to adult immunization. District A hosts two vaccination centers, while Districts B and C have one vaccination center each. The delivery of vaccination services is organized consistently across all districts, with both GPs and vaccination centers providing vaccination.

### 2.2. Study Design and Data Sources

This study is a retrospective observational cohort study considering all individuals residing in the LHA of Viterbo, Lazio region, Italy, who turned 65 years of age between 2018 and 2023.

For the construction of the dataset, data on individuals belonging to the birth cohorts of interest (1952–1958) were first extracted from the Regional Vaccination Registry (AVR), a digital platform for managing and recording vaccinations in the Lazio region. This initial dataset included the list of individuals along with summary information on the uptake of pneumococcal vaccination with the number of doses, but without information on the specific vaccine types that were administered. To clarify whether individuals who had received at least two doses were administered the sequential pneumococcal vaccination, a second internal dataset was retrieved containing details of pneumococcal vaccine types administered from 2018 to 2024 in the LHA of Viterbo, including the vaccination dates and the associated individuals. These two datasets were merged using a unique individual code as the key. All data were processed exclusively by LHA personnel as data controllers through procedures ensuring the confidentiality and security of the information, in compliance with EU Regulation 2016/679 (GDPR) and national legislation, based on consent obtained at the time of vaccination.

### 2.3. Variables Considered

The outcome variable—adherence to the sequential pneumococcal vaccination—was determined by evaluating these conditions:

1. The presence of at least two doses of pneumococcal vaccines registered in the AVR.
2. The registration of both PCV13 and PPSV23 in the internal database, with the latter administered after the PCV.
3. A time interval between the individual’s 65th birthday and an administration date of the PPSV23 vaccine of less than two years later.

The independent variables included sex, citizenship, district of residence, and municipality size, determined by linking ISTAT (National Institute of Statistics) population data to everyone’s place of residence.

### 2.4. Statistical Analysis

A descriptive analysis of the coverage in respect to the sequential pneumococcal vaccination was performed across cohorts of individuals turning 65 years old each year. Coverages were reported in percentages.

Additionally, a univariable analysis through the Chi square test and a logistic regression analysis was performed to assess the factors associated with the outcome variable. Independent variables included sex, citizenship, municipality size, and district of residence, and the results were presented as odds ratios (ORs) with 95% confidence intervals.

The significance level was set at 0.05 and statistical analysis were performed using RStudio (version 4.3.3).

### 2.5. Geomapping and Geocoding

Vaccination coverages were also geocoded using address information recorded in AVR. A geographic database with county and regional boundaries in vector format (SHP file) was obtained from Open Data Lazio Region (http://dati.lazio.it/catalog/it/dataset/limiti-amministrativi-dei-comuni-della-regione-lazio/resource/1985e1d3-fe26-4ee6-aca8-d1bd077f3b3b (accessed on 30 June 2025)). RStudio (version 4.3.3) was used for spatial analysis.

## 3. Results

The characteristics of individuals turning 65 years old across the whole study period and their vaccination coverage are summarized in Table 1. This cohort included a total of 27,657 individuals, of whom 640 completed the sequential pneumococcal vaccination, resulting in an overall coverage of 2.32%. Significant differences in vaccination coverage were identified with respect to all the independent variables considered. Females demonstrated a higher coverage (2.58%) compared to males (2.04%) (*p* = 0.003). Coverage also varied by citizenship, with Italian citizens exhibiting a higher coverage (2.45%) compared to foreign residents (0.64%). Across health districts, differences were observed, with District C showing the highest coverage (3.03%), followed by District B (2.76%) and District A (1.08%). Furthermore, the size of the municipality of residence was associated with significant differences, as individuals residing in municipalities with 10,000 or fewer inhabitants showed higher coverage (2.52%) compared to those in municipalities with more than 10,000 inhabitants (1.98%).

These differences resulted in an increased OR for the sequential pneumococcal vaccination in females compared to males (OR: 1.324, 95% CI: 1.13–1.553, *p* < 0.001), residents in municipalities with 10,000 or fewer inhabitants compared to those in larger municipalities (OR: 1.482, 95% CI: 1.233–1.786, *p* < 0.001), and residents in Districts B and C as compared to those in District A (OR: 3.074, 95% CI: 2.4–3.963 and OR: 2.822, 95% CI: 2.255–3.563, respectively, both *p* < 0.001) (Table 2). On the contrary, foreign citizens had substantially lower odds of completing the sequential pneumococcal vaccination compared to Italian citizens (OR: 0.245, 95% CI: 0.134–0.407, *p* < 0.001).

**Figure 2:**
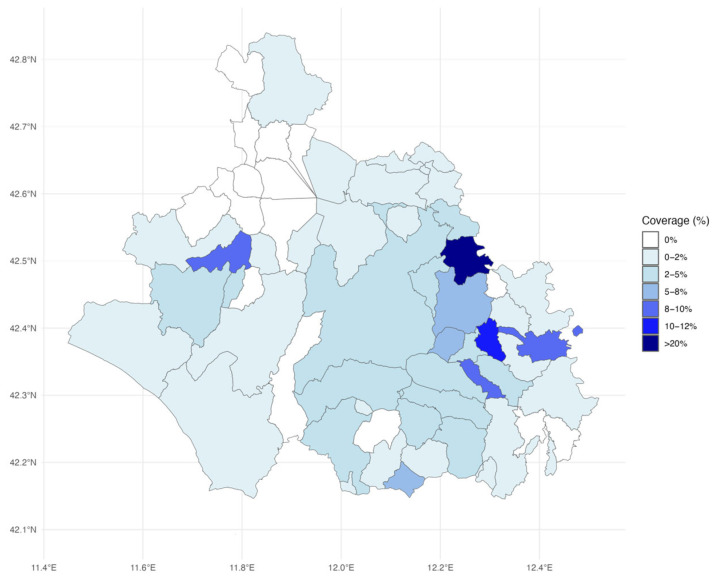
Coverage (%) across the municipalities of the Viterbo LHA.

The spatial analysis (Figure 2) highlights the highest vaccination coverage in municipalities in the eastern and southeastern regions, corresponding largely to District C. These municipalities were also characterized by smaller populations. In contrast, the northern region, which encompasses District A, exhibited lower coverage, with 10 municipalities recording coverage equal to 0.

Vaccination coverage among the newly 65-year-old residents in Viterbo LHA remained low from 2018 to 2023, with significant variability across years and subgroups (Table 3). In particular, coverage increased from 0.60% in 2018 to a peak of 3.27% in 2020, reflecting initial improvements in adherence to the vaccination campaign. However, this was followed by a plateau in 2021–2022 and a decline to 2.53% in 2023.

**Figure 3:**
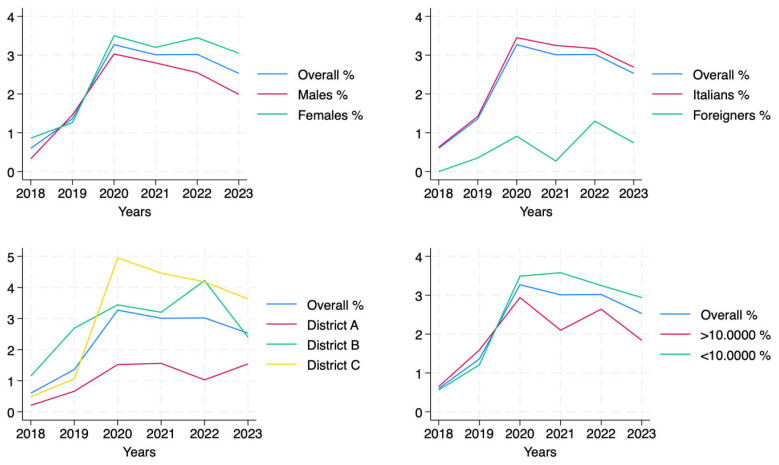
Comparison of sequential pneumococcal vaccination coverage trends across demographics and districts (2018–2023).

As illustrated in Figure 3, vaccination coverage trends among the newly 65-year-old residents showed variability across demographic and geographic subgroups. Females showed higher coverage than males, with an increase in the first few years and a moderate decline after 2020. Italian citizens followed a similar trend, maintaining higher coverage than foreign residents. Geographic analysis revealed that District C led in coverage, peaking at 4.72% in 2020, while District A had the lowest values, never exceeding 1.53%. Coverage was also higher in smaller municipalities (≤100,000 inhabitants), where the trend mirrored the overall population, with a marked rise in 2020, followed by a stabilization and subsequent decline.

## 4. Discussion

This retrospective observational cohort study examined the coverage of the sequential pneumococcal vaccination (PCV13 followed by PPSV23) among newly 65-year-old residents in Viterbo LHA between 2018, which was the year of the launch of the sequential vaccination strategy in the Lazio region, and 2023. The overall coverage across the whole study period was low, with improvements between 2018 and 2020 followed by a plateau and a decline.

Differences between demographic and geographical subgroups were also found. In particular, females, Italian citizens, and residents of smaller municipalities showed higher coverages. The district to which people belong also played a role.

Lower male adherence to the vaccination schedule is in line with previous studies on other vaccination coverages [24,25,26] and is consistent with the fact that women usually use healthcare services more than men [27,28]. Similarly, lower vaccination coverage among immigrants compared to Italian citizens has also been observed in previous studies, particularly in the context of COVID-19 and influenza vaccination programs [29,30,31,32].

The better performance observed in smaller municipalities is consistent with findings from other settings, where smaller municipalities tend to achieve higher vaccination coverage and lower dropout rates, possibly due to stronger community ties and more direct interaction with local health services [33,34]. However, behind the differences observed among municipalities and districts, there are likely broader structural and socioeconomic factors that may influence the uptake—such as education level, age, and employment [35]. Nevertheless, it was not possible to assess these aspects in our study due to the limitations of the administrative data used. Another aspect that needs to be considered is the challenge of reaching individuals with reduced autonomy. In this regard, although pneumococcal vaccination in Italy is typically delivered by General Practitioners (GPs), including through home visits when necessary, the main barrier may be less related to accessibility and more to the lack of a structured and consistently implemented recall system.

The observed decline seen after the peak in 2020 can be attributed to the impact of the COVID-19 pandemic, which disrupted health care services and vaccination campaigns worldwide. Nevertheless, after the pandemic, the introduction of PCV20 might also have contributed to the decline, as it has gradually replaced the sequential pneumococcal vaccination for newly 65-year-olds in Italy. Nevertheless, this effect concerns 2023 and, in the Lazio region, applies only to those not previously vaccinated, because those already vaccinated with PCV13 continue with the sequential schedule without serotype catch-up using PCV20.

The low coverage found in our study is in line with the evidence from the literature. In a recent systematic review [36], only five studies were found that reported data on the sequential schedule (PCV13 + PPSV23), with coverage ranging from < 1% in the Netherlands to 4.0% in Germany. Most of the available studies reported single-dose coverage with either PCV13 or PPSV23 with coverage ranging from 4.3% to 68.6%. Interestingly, the study on sequential vaccination conducted in Germany [37] addressed a high-priority group demonstrating the difficulty in achieving appropriate coverage, even in people who deserve to be vaccinated.

Information and education are two pillars that make it possible to increase vaccination coverage. The evidence [38,39] already provides clear proof of the effectiveness of the sequential pneumococcal vaccination. In respect to safety, a study conducted in Puglia [40] reported serious adverse events after PCV13 and PPSV23 in less than 0.1‰ of doses administered, with the vast majority being mild and self-limiting. Health professionals entrusted to deliver pneumococcal vaccination in the elderly, mostly represented in Italy by GPs, should take advantage of their relationship with the patients to provide them with accurate and reliable information about both the efficacy and the safety of the sequential pneumococcal vaccination. This is a fundamental step ing encouraging patients’ adherence to vaccination.

The observed limited vaccination coverage is also concerning considering that a recent cost-effectiveness analysis conducted in Italy [41] supports pursuing the sequential strategy independently based on the PCV used, because PPSV23 is affordable and increases health outcomes. If this is going to happen, it is necessary to develop and put in place interventions to increase the uptake of the second dose to protect people from pneumococcal diseases.

Strengths exist within this work. it gives a thorough assessment of the sequential pneumococcal vaccination coverage among newly 65-year-olds in a six-year time frame, revealing trends in vaccination and disparities in a natural setting. The findings of the study can be useful to inform public health interventions also based on differences shown across subgroups. Furthermore, the use of the regional vaccination registry as a source of data enhances the validity and reliability of the results.

However, there are also some limitations. The study did not collect data on potential health-related conditions that could impact the uptake of vaccination, thus preventing the analysis of coverage among high-risk subgroups. Additionally, the analysis considered people who completed the sequential vaccination within 2 years after their 65th birthday. Nevertheless, there may have been individuals who completed the sequential vaccination after this time window. Furthermore, the number of individuals in each cohort was based on the resident population at the start of follow-up, and no correction was applied for individuals who may have died during the two-year observation period. Similarly, as the study considered only the resident population in Viterbo LHA, it is possible that some individuals completed the vaccination outside the Lazio region. However, this limitation primarily applies to people being vaccinated in regions that have not yet implemented a regional immunization registry. For regions with an active registry, data are transmitted through the national immunization registry, which allows for cross-regional data integration. Considering all these issues, a possible underestimation of the coverage cannot be excluded, albeit the overall impact on the estimate is expected to be limited. Individual reasons for not being vaccinated were not evaluated, leaving some gaps in understanding potential barriers to pneumococcal vaccination.

Another relevant aspect that emerged was the gap in completeness of data in the AVR, which was overcome by consulting another internal database. This can limit the transferability of our approach to other contexts. Finally, our findings—in particular, those regarding the geographical disparities—cannot be considered generalizable because they are strictly related to the Viterbo LHA organizational context. In fact, although the districts share the same organization of vaccination delivery, differences that were observed could be explained by GP participation in the campaign and logistical access issues.

These limitations suggest the need for additional research to create a more holistic understanding of pneumococcal vaccination uptake.

## 5. Conclusions

The present study highlighted that the coverage of the sequential pneumococcal vaccination has remained very low after the launch of the strategy. Furthermore, significant differences emerged in respect to individual and geographical characteristics. This underscores a significant gap between recommendations and their actual implementation.

Monitoring vaccination coverage and also assessing inequalities allows for the implementation of public health strategies to address potential criticisms. The introduction of electronic vaccination registries has clearly elevated the capacity to monitor coverage with secure and timely access to data. Nevertheless, resources and methods should be implemented to routinely carry this out. Furthermore, the assessment of coverage cannot be complete without the addition of other important information, like patients’ health status or medical indications/contraindications for vaccination. While these data are typically requested when a patient is registered for the vaccine, they are often not complete, meaning they remain mostly unusable.

Improving the completeness and accuracy of data would enable health authorities to better understand how the vaccination strategy is deployed and adjust their approaches to suit various population groups’ needs, ultimately enhancing the success of vaccination campaigns.

## Figures and Tables

**Table 1:** Characteristics of the newly 65-year-old population and sequential pneumococcal vaccination coverage, Viterbo LHA (2018–2023).

Variables	Vaccinated	Newly 65-Year-Old Population (%)	Coverage %(IC95)	*p*-Value
Total	640	27,657 (100)	2.32 (2.10–2.50)	
Gender				
F	365	14,158 (51.2)	2.58 (2.32–2.85)	0.003
M	275	13,499 (48.8)	2.04 (1.81–2.29)	
Citizenship				
Italian	627	25,637 (92.7)	2.45 (2.26–2.64)	<0.001
Foreigner	13	2020 (7.3)	0.64 (0.34–1.10)	
District				
A	98	9048 (32.72)	1.08 (0.88–1.32)	<0.001
B	223	8069 (29.18)	2.76 (2.42–3.15)	
C	319	10,540 (38.1)	3.03 (2.71–3.37)	
N inhabitants in the municipality of residence				
>10,000	212	10,688 (38.65)	1.98 (1.73–2.27)	0.004
≤10,000	428	16,969 (61.35)	2.52 (2.29–2.77)	

**Table 2:** Logistic regression results: factors associated with completion of pneumococcal sequential vaccination.

Variables	OR	IC95%	*p*-Value
Gender (ref. Male)	1.324	(1.13–1.553)	<0.001
Citizenship (ref. Italian)	0.245	(0.134–0.407)	<0.001
N inhabitants’ municipality of residence (ref. >10,000)	1.482	(1.233–1.786)	<0.001
District B District C (ref. District A)	3.074 2.822	(2.4–3.963) (2.255–3.563)	<0.001 <0.001

**Table 3:** Pneumococcal sequential vaccination coverage across years and variables, Viterbo LHA (2018–2023).

Years		Vaccinated	Newly 65-Year-Old Population	Coverage (%)
2018	Total	26	4351	0.60
	Female	19	2209	0.86
	Male	7	2142	0.33
	Italian	26	4109	0.63
	Foreigner	0	242	0.00
	District A	3	1404	0.21
	District B	15	1316	1.14
	District C	8	1631	0.49
	>10,000	11	1685	0.65
	≤10,000	15	2666	0.56
2019	Total	63	4638	1.36
	Female	30	2374	1.26
	Male	33	2264	1.46
	Italian	62	4356	1.42
	Foreigner	1	282	0.35
	District A	3	1404	0.21
	District B	15	1316	1.14
	District C	8	1631	0.49
	>10,000	28	1757	1.59
	≤10,000	35	2881	1.21
2020	Total	150	4588	3.27
	Female	82	2341	3.50
	Male	68	2247	3.03
	Italian	147	4258	3.45
	Foreigner	3	330	0.91
	District A	22	1466	1.50
	District B	46	1384	3.32
	District C	82	1738	4.72
	>10,000	55	1869	2.94
	≤10,000	95	2719	3.49
2021	Total	140	4658	3.01
	Female	77	2405	3.20
	Male	63	2253	2.80
	Italian	139	4281	3.25
	Foreigner	1	377	0.27
	District A	24	1567	1.53
	District B	42	1356	3.10
	District C	74	1735	4.27
	>10,000	38	1807	2.10
	≤10,000	102	2851	3.58
2022	Total	141	4670	3.02
	Female	84	2435	3.45
	Male	57	2235	2.55
	Italian	136	4285	3.17
	Foreigner	5	385	1.30
	District A	16	1567	1.02
	District B	53	1308	4.05
	District C	72	1795	4.01
	>10,000	47	1781	2.64
	≤10,000	94	2889	3.25
2023	Total	120	4752	2.53
	Female	73	2394	3.05
	Male	47	2358	1.99
	Italian	117	4348	2.69
	Foreigner	3	404	0.74
	District A	23	1518	1.52
	District B	33	1406	2.35
	District C	64	1828	3.50
	>10,000	33	1789	1.84
	≤10,000	87	2963	2.94

## Data Availability

The data presented in this study are available on request from the corresponding author. The data are not publicly available as they are the property of the Local Health Authority (ASL) of Viterbo, and any request for access must be evaluated and authorized by the ASL.
